# Etiological Work-Up for Adults with Bronchiectasis: A Predictive Diagnostic Score for Primary Ciliary Dyskinesia and Cystic Fibrosis

**DOI:** 10.3390/jcm10163478

**Published:** 2021-08-06

**Authors:** Frederic Schlemmer, Agnes Hamzaoui, Sonia Zebachi, Aurelie Le Thuaut, Gilles Mangiapan, Isabelle Monnet, Amel Boudjema, Laurence Jabot, Bruno Housset, Sylvie Bastuji-Garin, Laurence Bassinet, Bernard Maitre

**Affiliations:** 1 Service de Pneumologie, Centre Hospitalier Intercommunal, FHU SENEC, F-94000 Créteil, France; gilles.mangiapan@chicreteil.fr (G.M.); isabelle.monnet@chicreteil.fr (I.M.); amel.boudjemaa@chicreteil.fr (A.B.); laurence.jabot@chicreteil.fr (L.J.); bruno.housset@chicreteil.fr (B.H.); kilad@orange.fr (L.B.); bernard.maitre@chicreteil.fr (B.M.); 2 Unité de Pneumologie, AP-HP, Hôpitaux Universitaires Henri Mondor, FHU SENEC, F-94010 Créteil, France; 3 Université Paris Est Créteil, INSERM, IMRB U955, F-94010 Créteil, France; 4 Unité de Recherche (UR/12SP15), Départment des Maladies Respiratoires, Division de Pneumologie, Hôpital Abderrahman Mami, Ariana 2080, Tunisia; agnes.hamzaoui@gmail.com; 5 Département de Santé Publique, AP-HP, Hôpitaux Universitaires Henri Mondor, FHU SENEC, F-94010 Créteil, France; sonia.zebachi@aphp.fr (S.Z.); aurelie.le-thuaut@aphp.fr (A.L.T.); sylvie.bastuji-garin@aphp.fr (S.B.-G.)

**Keywords:** bronchiectasis, cystic fibrosis, primary ciliary dyskinesia

## Abstract

Background: etiological investigations are not done for all adult patients with bronchiectasis because of the availability and interpretation of tests. The aim of the study was to elaborate a score to identify patients at high risk of having cystic fibrosis or primary ciliary dyskinesia (CF/PCD), which require appropriate management. Methods: diagnostic work-ups were carried out on a French monocenter cohort, and results were subjected to logistic-regression analyses to identify the independent factors associated with CF/PCD diagnosis and, thereby, elaborate a score to validate in a second cohort. Results: among 188 patients, 158 had no obvious diagnosis and were enrolled in the algorithm-construction group. In multivariate analyses, age at symptom onset (8.69 (2.10–35.99); *p* = 0.003), chronic ENT symptoms or diagnosed sinusitis (10.53 (1.26–87.57); *p* = 0.03), digestive symptoms or situs inversus (5.10 (1.23–21.14); *p* = 0.025), and Pseudomonas. aeruginosa and/or Staphylococcus aureus isolated from sputum (11.13 (1.34–92.21); *p* = 0.02) are associated with CF or PCD. Receiver operating characteristics curve analysis, using a validation group of 167 patients with bronchiectasis, confirmed the score’s performance with AUC 0.92 (95% CI: 0.84–0.98). Conclusions: a clinical score may help identify adult patients with bronchiectasis at higher risk of having CF or PCD.

## 1. Introduction

Bronchiectasis is a lung condition involving abnormally dilated, thick-walled bronchi that are chronically inflamed and infected by bacteria. This disease frequently results in notable physical and social morbidity. Treatment is generally symptom-oriented to limit progressive functional loss. Identification of the cause may lead to management changes, thereby improving treatment efficacy, obtaining a more precise prognosis estimation and allowing for genetic counselling [1]. Appropriate management is crucial for three specific diseases: primary ciliary dyskinesia (PCD), cystic fibrosis (CF) and common variable immunodeficiency (CVID).

The British Thoracic Society (BTS) and European Respiratory Society (ERS) Bronchiectasis Task Force proposed a minimum etiological test bundle for adults with newly diagnosed bronchiectasis: differential blood count, serum immunoglobulins (total IgG, IgA and IgM) and allergic bronchopulmonary aspergillosis (ABPA) testing [2,3]. Detailed investigations are usually carried out in response to clinical findings and access to advanced testing facilities, but no standardized criteria exist to identify patients needing an extensive work-up. Both Societies also suggested excluding CF for young adults or with evocative clinical features, e.g., predominantly upper lobe predominance on chest computed-tomography (CT) scans, nasal polyposis and/or chronic rhinosinusitis, recurrent pancreatitis, male primary infertility and/or malabsorption. According to those proposals and the ERS Task Force for PCD diagnosis [4], specific testing should be considered for patients with several of the following features: persistent wet cough since childhood, situs anomalies, congenital cardiac defects, nasal polyposis and/or chronic rhinosinusitis, chronic middle ear disease, a history of neonatal respiratory distress or neonatal intensive care unit admission of term infants. However, the evidence levels of such proposals has generally been low.

Moreover, the required laboratory competencies and result-interpretation skills, as well as the inconvenience and cost of investigations, may be substantial and may explain, for the most part, why this etiological work-up is not done for all patients. In a preliminary report from the European Bronchiectasis Registry (EMBARC; unpublished results), <40% of patients have a minimum of investigations (white blood cell count, immunoglobulin levels, ABPA screening). Unsurprisingly, a survey of 26 European countries found that PCD is underdiagnosed, particularly in countries with low healthcare expenditures [5]. 

This study was undertaken to ascertain the clinical, biological and imaging features of a French monocenter adult bronchiectasis cohort, according to underlying etiology, with the aim of elaborating an algorithm able to detect two specific diagnoses—CF and PCD—that require management changes and necessitate complex laboratory analyses. Our final goal was to try to reduce the number of patients requiring an extensive work-up in this context.

Several clinical and biological factors are associated with CF/PCD in adults: chronic ENT disorders, digestive symptoms, dextrocardia and Staphylococcus aureus and/or Pseudomonas aeruginosa isolation from sputum. A score was created with a construction cohort and validated on another cohort.

## 2. Materials and Methods

### 2.1. Patients

Adults with chronic respiratory symptoms were routinely referred to our specialized tertiary structure. Bronchiectasis was diagnosed on thoracic high-resolution CT (HRCT) scans of patients with compatible clinical findings. Patients with active lung cancer, known CF, COPD or interstitial lung diseases were not included. Two patient groups were recruited prospectively. 

All patients gave their informed oral consent for the clinical work-up. Written consent was obtained for genetic testing. The study was approved by the Research Ethics Committee of the Société de Pneumologie de Langue Française. Patients’ complete medical histories were recorded, including age at pulmonary symptom onset; neonatal respiratory distress; age at bronchiectasis diagnosis; family history (asthma, bronchiectasis, rhinosinusitis, infertility); smoking; childhood respiratory infections, focusing on measles and pertussis, severe pneumonia, pulmonary tuberculosis and asthma; and extra-respiratory manifestations, including gastrointestinal symptoms (chronic diarrhea, steatorrhea, constipation) and infertility. Chronic ear, nose and throat (ENT) disorders include all persistent or recurrent symptoms and disorders (persistent purulent rhinosinusitis, sinus surgery, otitis, nasal polyposis, conductive hearing loss) except allergic rhinitis (or suggestive symptoms, e.g., aqueous rhinorrhea, nasal pruritus, and pharyngeal pruritus). Gastroesophageal reflux (GERD) manifestations were also noted. Chronic associated diseases were considered comorbidities. A pulmonologist and an ENT specialist conducted the physical examination.

Pulmonary function tests (PFTs): FEV1, FVC, TLC and blood gases were measured according to ERS/ATS recommendations [6]. Best values during the first year of follow-up were recorded.

Microbiological studies included repeated sputum examinations and cultures (usual bacteria, fungi and mycobacteria). A patient was considered colonized with bacteria if he/she had positive cultures on at least three occasions, at least three months apart, over a one-year period [1]. 

When bronchoscopy was used to look for luminal abnormalities, because of hemoptysis or to examine cilia in a bronchial biopsy or brushing, microbiological samples were also obtained. 

Imaging included thoracic and nasal sinus HRCT scans. Based on a modified Bhalla score, the bronchiectasis extent, severity and bronchial wall thickness were evaluated for each lobe, and summed according to the number of pulmonary lobes and segments affected, with the lingula and middle lobe considered independently [7]. Bronchiectasis location, saccular lesions and associated abnormalities were also recorded.

### 2.2. Etiological Work-Up

All patients underwent the same standardized etiological work-up. The laboratory work-up was extended to look for humoral immunodeficiencies (serum immunoglobulin levels, IgG subclasses quantified by standardized nephelometric assays), serum α1-antitrypsin level, human immunodeficiency virus (HIV), anti-Aspergillus fumigatus antibodies (Paragon^®^ Immunoelectrophoresis, Beckman Coulter, France) and autoantibodies. When a patient reported asthma symptoms, total serum IgE and specific anti-Aspergillus fumigatus IgE levels were measured (CAP system/unicap, Pharmacia, Bethesda, MA, USA). All patients also had the following investigations:

(1) A sweat chloride test according to the recommendations [8]. A trans-epithelial nasal potential difference was reserved for those with dubious results from the sweat chloride test [9]. A screening for CF transmembrane-conductance regulator gene (CFTR)-mutation (OLA Cystic Fibrosis Assay, Abbott, IL, USA) was performed when the sweat chloride test was abnormal.

(2) An examination of ciliated cells, obtained by nasal or bronchial brushing. In addition, since 2004, nasal nitric oxide (NO) has also been quantified [10].

A nasal or bronchial mucosa biopsy was performed and subjected to electron microscopy (EM) when cytology and/or NO results were suggestive of PCD.

Etiological diagnoses were reached collegially (by four chest physicians: FS, LB, AH and BM) based on the etiological test results. Dubious or conflicting diagnoses were considered undefined. Specific diagnoses were made according to international criteria. Diagnosis of CF was made according to the combination of clinical symptoms, sweat chloride test results and genetic analysis according to [8]. PCD diagnosis was made according to ERS task force recommendations [4].

### 2.3. Data Collection, Analysis and Statistics

Categorical variables are expressed as numbers (%), and quantitative variables as mean ± SEM or median (IQR), when specified.

### 2.4. CF/PCD Diagnosis Score Construction

We considered the etiological factors described above as essential to diagnose CF, PCD and humoral immunodeficiencies because of their potential impacts on patient management and genetic counseling. Because we think that CF and PCD share similar clinical symptoms, we examined the characteristics associated with CF and PCD (CF/PCD) diagnosis.

First, we eliminated the obvious diagnoses (inflammatory diseases and extrinsic factors) for which no further or only limited evaluation was necessary. Then, we excluded humoral immunodeficiencies, because these tests are widely used and readily available.

The characteristics of patients with a CF/PCD diagnosis and without such a diagnosis were compared using χ^2^, Fisher’s exact or Wilcoxon–Mann–Whitney tests, as appropriate. Odds ratios (ORs) (95% confidence interval (CI)) were estimated using univariate logistic-regression models. Variables achieving univariate *p* < 0.15 were entered into the multivariate model. Confounders and interactions were assessed in bivariate models. To avoid introducing strongly correlated variables into multivariate models, we assessed correlations using Cramer’s V for categorical variables and the nonparametric Spearman’s rank correlation for quantitative variables (Rho); values >0.50 were taken to indicate strong correlations. Age at symptom onset was dichotomized at 15 years for multivariate analyses. The multivariate model β-coefficients were used to develop the algorithm-scoring system. To validate the score internally, 1000 bootstrap replications were used to estimate shrinkage β-coefficients [11]. The doubled, median, bootstrapped regression coefficients rounded to the nearest integer served as weights that were combined with the baseline logit function to derive the prediction score.

Multivariate model and score discrimination and calibration were assessed. Discrimination was assessed using the C statistic with bootstrapped 95% CIs (area under the receiver operating characteristics (ROC) curve, AUC), with the usual thresholds (≥0.7, good; ≥0.8, very good; and ≥0.9, excellent discrimination); calibration was evaluated by computing the Hosmer–Lemeshow χ^2^ statistic (*p* > 0.20, good fit).

We calculated the sensitivity, specificity, likelihood ratios and percentage of correctly classified subjects for each prediction-score value to determine the optimal threshold.

### 2.5. Model Validation

Score performance, including calibration and discrimination, was assessed with the second validation cohort.

All tests were two-tailed. No imputation for missing values was performed. Data were analyzed using STATA software version 14 (Stata Inc., College Station, TX, USA).

## 3. Results

### 3.1. Patients’ Characteristics

**Figure 1:**
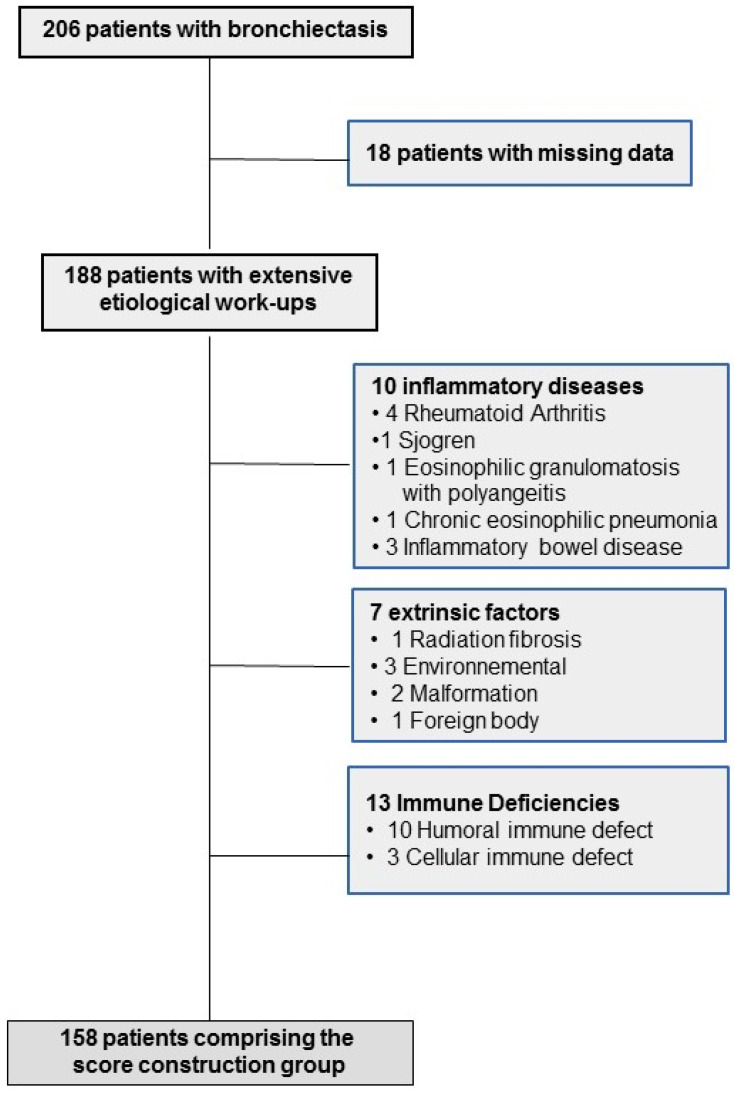
Study flow chart for the construction cohort.

Among 206 adult patients with bronchiectasis who were recruited, 18 with missing data were immediately excluded, leaving 188 who underwent extensive etiological work-ups; their characteristics are reported in online Supplementary Table S1. After excluding the patients with obvious diagnoses, 158 patients (median age 57.5 years; mostly females) comprised the score construction group (Figure 1).

Symptom onset preceded bronchiectasis diagnosis by ~10 years. Childhood respiratory infections had been very common, and 50% had a family history of respiratory diseases. Fourteen patients had previously undergone thoracic surgery: 11 for bronchiectasis, at a mean age of 20 years, and the left lower lobe had been removed from more than half.

For the 158 patients, respiratory symptoms included wheezing (23.8%), chronic cough (82.8%), recurrent infections (79.5%), hemoptysis (41.1%) and dyspnea (69.5%). ENT diseases, particularly sinusitis, were frequent (56.3%), and sinus CT scans revealed sinusitis (45.1%) and polyps (10.6%). GERD manifestations were present in 34.5%. Comorbidities, reported in 60.3% of the patients, increased with age (e.g., 15.2% with cancer), and were also associated with smoking. On imaging, upper lobes were less frequently involved than middle and lower lobes. Bronchiectasis was bilateral in 84.1% of the patients, with saccular lesions seen in 28.5%. Localized bronchial abnormalities were observed by imaging in 13%. Pseudomonas aeruginosa (PA), Staphylococcus aureus (SA), Streptococcus pneumoniae and Haemophilus influenzae were found, respectively, in 29.2%, 9.3%, 10% and 8%, and non-tuberculosis mycobacteria in 9.3%. A total of 19.2% were PA-colonized and 34.2% had no identified bacteria. PFTs indicated an obstructive pattern in 67.5% of patients and a restrictive defect in 17.9%, with 11.6% having a mixed pattern. The cohort’s median ± IQ FEV1 was 72 [17; 91] of the predicted value.

### 3.2. Etiologies

Underlying etiologies were identified for 135 (71.8%) patients. For 37 (19.7%), the identified cause (CF, PCD, immunodeficiency or α1-antitrypsin deficiency) led to distinct and individualized management change(s) and/or genetic counselling.

Humoral immunodeficiencies included three CVID, six IgG-subclass deficiencies and one IgG + IgM deficiency. CF patients’ characteristics are given in online Supplementary Table S2.

PCD was diagnosed in 12 patients, and 4 had situs inversus. Ciliary beat frequency (CBF) was <8 Hz in all but one, who had abnormal high-speed video microscopy beating. Nasal NO was <50 nL/min in 6 patients, not feasible for 3 patients with poor lung function, and not performed for 3 patients. Electronic microscopy results were abnormal in 7 patients, normal in 1 patient with situs inversus and unfeasible for 4 patients. Genetic analysis showed identification of two nonambiguous mutations in known PCD genes in these last five patients.

### 3.3. Model Development

Univariate analyses of factors potentially associated with CF/PCD diagnosis (Table 1) found that all variables, except HRCT scoring, bronchiectasis location, PFTs and sex, were significantly associated with the diagnosis.

Because of the strong correlations observed among age parameters, namely age, age at bronchiectasis diagnosis, age at symptom onset, and number of patients with age at symptom onset <15 years (Rho > 0.57), we considered only early symptom onset (<15 years) for multivariate analysis. Due to the weak number of patients with dextrocardia or digestive symptoms, we regrouped these variables to one item, labelled extra respiratory involvement. According to multivariate analyses, four variables, namely early onset, chronic ENT disorders, digestive symptoms and/or dextrocardia, PA and/or SA isolation, remained independently associated with CF/PCD diagnosis (Table 2). After adjustment for chronic ENT disorders and PA and/or SA isolation, family history of bronchiectasis and digital clubbing were no longer associated with CF/PCD diagnosis. The model had adequate calibration (*p* = 0.32) and excellent discrimination (ROC AUC = 0.91 (95% CI: 0.86–0.98)).

The CF/PCD diagnosis algorithm score was computed by means of a linear combination of the doubled bootstrapped regression β-coefficients rounded to the nearest integer, as follows: 5 × (early onset) + 3 × (chronic ENT disorders) + 4 × (digestive symptoms and/or dextrocardia) + 5 × (SA and/or PA isolation). The algorithm score (range: 0–17) was then used in a new logistic equation to estimate the probability of CF/PCD diagnosis. This model is described by the following formula: logit = −4.94 + 0.48(CF/PCD score). The score’s calibration was adequate (*p* = 0.34), and its discrimination very good (ROC AUC = 0.89 (95% CI: 0.76–0.95)). A cut-off of 8 yielded 90.9% sensitivity, 82% specificity and 98.1% NPV.

### 3.4. Score Validation

The second group of 167 patients with bronchiectasis (validation cohort), recruited between October 2007 and December 2011, was used to validate the constructed algorithm score. This evaluation analyzed whether the score’s predictive capacity for the validation cohort and its division into etiological groups showed any significant differences when compared with the same analyses using the data from the construction cohort.

The validation group’s female/male ratio was 0.57 and mean age was 60.8 ± 17.2 (range: 20–92) years. Mean age at symptom onset was 41.7 ± 23.4 (range: 0–48) years. Diagnoses were identified for 117 (70.3%) patients (Table 3). After excluding the 22 obvious diagnoses and nine immune deficiencies, scores were calculated for the 136 remaining patients. The agreement between the observed (10.3%) and expected (10.2%) percentages of patients diagnosed with CF/PCD was good (calibration, *p* = 0.67). ROC-curve analysis confirmed the score’s performance, with AUC = 0.92 (95% CI: 0.84–0.98).

### 3.5. Performance of PCD Scores in Validation Cohort

**Figure 2:**
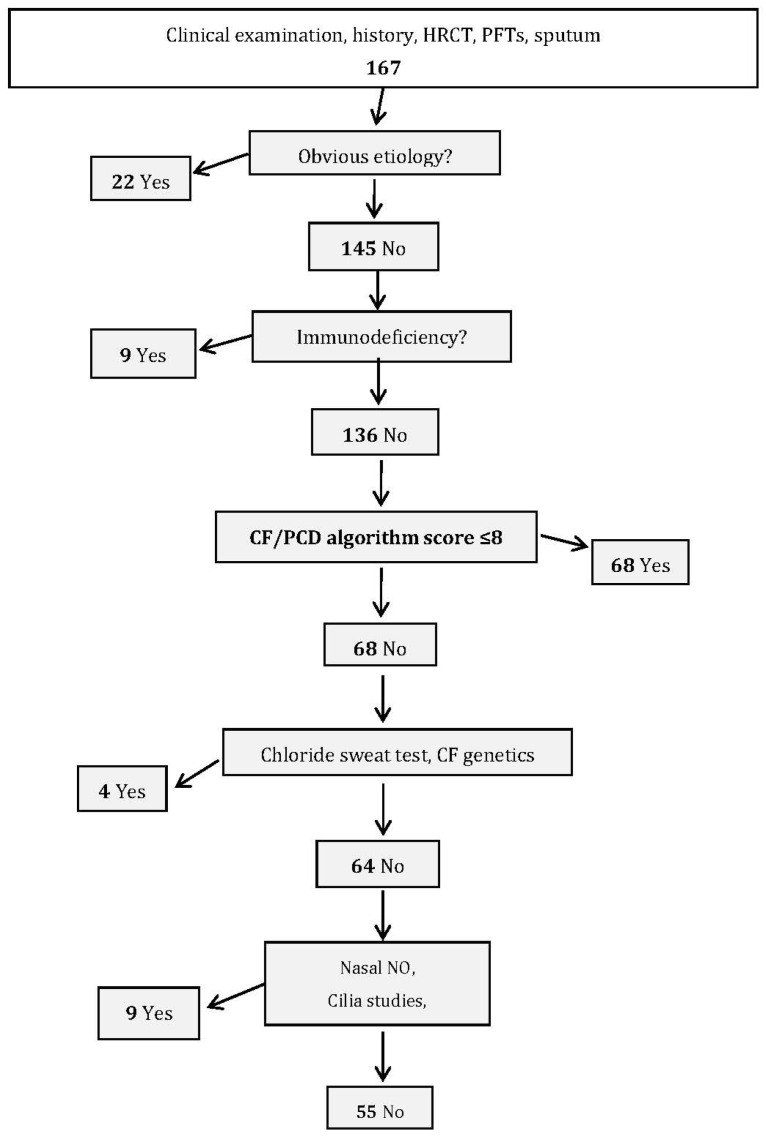
Proposed etiological work-up algorithm to diagnose CF or PCD. CF, cystic fibrosis; PCD, primary ciliary dyskinesia; HRCT, high-resolution computed-tomography scan; PFTs, lung function tests in the validation cohort.

Since different predictive diagnostic tools have been described in the literature (PICADAR score and “Leigh” score) for determining the likelihood of PCD in children and adults, we calculated these two scores in our validation cohort. As shown in Table S3, these two tests were not able to discriminate patients with PCD in our cohort. Using our score might halve the number of patients undergoing specific tests for CF and PCD in the validation study (Figure 2).

## 4. Discussion

In this monocenter study on adults with bronchiectasis, an etiology was identified in 72% for the construction cohort and 70.3% of the validation group, after eliminating patients with obvious diagnoses (inflammatory diseases, extrinsic factors) and immunodeficiencies. We then built a composite CF/PCD diagnosis algorithm and used age of symptom onset <15 years, chronic ENT disease, diagnosed sinusitis, digestive symptoms, dextrocardia and PA and/or SA isolation from sputum to score these clinical variables. The score was validated on the second patient cohort.

To our knowledge, no French study on bronchiectasis patients has applied a systematic work-up to define etiology. Our results are similar to those of previous studies, particularly a 2010 large European study [12]. We found frequencies of 35% post-infectious disease and 22.8% idiopathic bronchiectasis and, compared to the latter study, fewer of our patients had COPD (1.1 vs. 15%), probably because our inclusion criteria did not include patients with previously diagnosed bronchiectasis-associated lung diseases. Their exclusion might explain our cohort’s younger mean age compared to that European study [12].

The authors of recent publications have suggested that a mandatory etiology work-up would decrease the number of idiopathic bronchiectasis diagnoses [12,13]. However, clinically suspected CF or PCD was not well-described in these studies. For ERS, testing should be considered for patients with several of the following features: persistent wet cough since childhood, situs anomalies, congenital cardiac defects, nasal polyposis and/or chronic rhinosinusitis, chronic middle ear disease with or without hearing loss, and a history of neonatal respiratory distress or neonatal intensive care admittance in term infants. For the BTS guidelines, PCD was ruled out only when recurrent sinusitis and/or chronic otitis media were present, or when a history of symptoms is present, including symptoms since childhood, childhood chronic otitis media, predominantly middle lobe bronchiectasis, infertility or dextrocardia [2,3]. Suggestions regarding CF diagnosis did not focus on clinical symptoms, but rather were focused on performing CF tests in young patients (<40 years) and/or patients with “specific” features, e.g., upper lobe bronchiectasis predominance on chest CT scans, nasal polyposis and/or chronic rhinosinusitis, recurrent pancreatitis, persistent isolation of Staphylococcus aureus in the sputum, male primary infertility and/or malabsorption.

In this context, we undertook extensive work-ups to devise our score and herein propose a three-step etiological work-up, based on disease frequency, available laboratory competences and result-interpretation perspicacity, and inconvenience and cost of investigations: (1) identify obvious etiologies based on history, imaging and minimal laboratory work-up (serum α1-antitrypsin level, HIV-infection testing, anti-*Aspergillus* antibodies, autoantibodies, total serum IgE); (2) determine serum immunoglobulin and IgG-subclass concentrations to diagnose immunodeficiencies; (3) restrict to patients with high probability of CF/PCD diagnosis, based on the CF/PCD algorithm score. The chloride sweat test and CF genetics are to be run first, and if normal, nasal NO level, cilia study and PCD genetics may be tested. For our cohort, algorithm implementation and score calculation would halve the percentage of patients requiring CF and PCD tests (Figure 2).

Recently, two diagnostic tools were proposed to predict PCD. Behan et al. recruited a multicenter English cohort of adults patients to create the PICADAR score [14]. The construction cohort comprised 641 patients, among whom 75 had a final PCD diagnosis. The validation group was a random selection of 157 patients, half of whom had a final PCD diagnosis. The items included in the PICADAR score are as follows: full-term delivery, chest symptoms during the neonatal period, hospitalization in a neonatal care unit, situs abnormality, congenital heart defect, persistent perennial rhinitis and chronic ear or hearing symptoms. Application of that score to a population of adults with bronchiectasis is limited,, because the score’s construction group combined children and adult data, and the patients’ ages at diagnosis were not known; in addition, the validation group included only children with a high PCD frequency. Another recently published multicenter study [15] from nine North American groups enrolled 534 children, among whom 205 had definite PCD. Similar items were found: laterality defect, unexplained neonatal respiratory distress, nasal congestion and cough. A publication focused on adult patients also proposed a PCD score using the modified PICADAR score items associated with nasal NO measurements [16].

In our cohort, the PICADAR and “North American” scores are not able to screen patients with high suspicion of PCD. For our adult cohort, the possibly surprising absence of neonatal respiratory distress as one of our factors can be explained in several ways. First, this item relies on early life information, which is often difficult to recall in adulthood. Second, we could expect that patients with neonatal respiratory distress and ENT and lung symptoms might have been screened for PCD and diagnosed before adulthood. Notably, only 23% of the 78 adult PCD patients that we previously described had neonatal respiratory distress [17]. In the same way, although infertility and history of consanguinity are features associated with PCD and CF, these two parameters were not statistically different between CF/PCD and non CF/PCD patients. This is probably explained by the low number of patients involved in our study.

Criteria for CF diagnosis are based mainly on laboratory tests (abnormal sweat chloride test, ion-transport abnormalities across the nasal epithelium or identification of a mutation in each CFTR gene) and one suggestive clinical symptom (among persistent respiratory infections with mucoid PA or Burkholderia cepacia, bronchiectasis in both upper lobes, nasal polyps and/or congenital bilateral absence of the vas deferens) or suggestive but less-specific clinical manifestations (various gastrointestinal and/or sinopulmonary signs, digital clubbing, atypical diabetes and/or osteopenia/osteoporosis <40 years of age). Based on these analyses, the phenotypes of patients with intermediate sweat-chloride values according to the European consensus statement to differentiate patients with CF, CFTR-related disease or unlikely CF [18], suggested that clubbing, SA or PA isolation and recurrent lower respiratory tract infections were more frequent in CF or CFTR-related disease. To our knowledge, no score has been created in the context of a bronchiectasis diagnosis in adults. Despite the easy access to CF diagnosis tests, we have observed that patients with symptoms very suggestive of CF in childhood had a CF diagnosis only in adulthood. This emphasizes the need for a clinical score.

A common sinopulmonary syndrome in CF and PCD and the small number of patients rendered their separation difficult. Therefore, we decided to create a combined CF/PCD diagnosis algorithm and weighted score.

Limitations of our study include the small number of patients with PCD and CF as well as the single tertiary-center recruitment. Although situs inversus is included in the PICADAR and our scores, its association with bronchiectasis is highly suggestive of PCD and, hence, might not necessarily be included in a score. Since almost 50% of PCD patients had dextrocardia, a very large cohort will be needed to establish a score without this item. The study’s strengths are that all patients from a large bronchiectasis cohort underwent the same systematic etiological work-up by a team experienced in diagnosing PCD and CF and that our study is focused on adult patients without previous diagnosis in childhood.

To summarize, our results highlighted several clinical and biological factors associated with CF/PCD diagnoses. We were able to devise an algorithm and weighted score, which may help select patients at high probability of having CF or PCD. We propose a sequential three-step algorithm for the etiological work-up of adults with bronchiectasis. Applying this score may markedly lower the number of patients requiring testing for CF/PCD. Since the number of patients with CF or PCD diagnosis is small in our study, a large multicenter study is warranted to validate this approach.

## Figures and Tables

**Table 1:** Comparison of CF/PCD and non-CF/PCD adult patients using univariate analyses (*n* = 158).

Characteristic	Overall *n* = 158	CF/PCD *n* = 22 (13.9)	Non-CF/PCD *n* = 136 (86.1)	*p* Value ^†^	OR (95% CI) *	*p* Value
Clinical						
Male	50 (31.6)	7 (31.8)	43 (31.6)	0.985	1.01 (0.38–2.65)	0.98
Age, median (Q1–Q3), years	57.5 (44–71)	38 (30–60)	60 (48.5–71.5)	<0.001	0.94 (0.92–0.97)	<0.001
Age at bronchiectasis diagnosis, median {Q1–Q3}, years	46.5 (27–61)	23 (12–30)	49.5 (33.5–62)	<0.001	0.95 (0.92–0.97)	<0.001
Age at symptom onset, median {Q1–Q3}, years (*n* = 150)	32 (6–55)	6 (1–12)	36 (6.5–57)	<0.001	0.93 (0.89–0.96)	<0.001
<15 years	55 (36.7/34.8)	19 (86.4)	36 (28.1/26.5)	<0.001	16.18 (4.51–58.05)	<0.001
Family bronchiectasis (*n* = 156)	14 (9.0/8.8)	6 (27.3)	8 (6.0/5.9)	0.006	5.91 (1.82–19.21)	0.003
Chronic ENT disorders	86 (54.4)	21 (95.4/5)	65 (47.8)	<0.001	22.94 (3.0–175.4)	0.003
Digital clubbing	5 (3.2)	3 (13.6)	2 (1.5)	0.020	10.58 (1.66–67.45)	0.01
Digestive symptoms	20 (12.7)	7 (31.8)	13 (9.6)	0.004	4.41 (1.52–12.79)	0.006
Dextrocardia	4 (2.5)	4 (18.2)	0 (0)	<0.001	–	
Digestive symptoms and/or dextrocardia	23 (14.5/6)	10 (45.4/5)	13 (9.6)	<0.001	7.88 (2.86–21.76)	<0.001
Bacterial isolation						
*P. aeruginosa* and/or *S. aureus* isolation						
None	102 (64.6)	7 (31.2)	95 (69.8/9)		Referent	–
*P. aeruginosa* or *S. aureus*	45 (28.5)	7 (31.8)	36 (26.5)	<0.001	3.39 (1.17–9.8)	0.007
*P. aeruginosa* and *S. aureus*	11 (7)	6 (27.3)	5 (3.7)		16.28 (3.96–66.93)	<0.001
Spirometry, median (Q1–Q3)						
FEV_1_, % (*n* = 158)	74 (56–92)	75 (56–90)	73.5 (55.5–92.5)	0.994	1.00 (0.98–1.02)	0.939
FVC, % (*n* = 137)	93 (74–107)	93 (79–110)	93 (74–106)	0.972	0.99 (0.98–1.02)	0.844
PaO_2_ (mm Hg) (*n* = 142)	83 (74–94)	83 (67–99)	82 (75–92)	0.989	0.99 (0.96–1.01)	0.313
PaCO_2_ (mm Hg) (*n* = 142)	39 (35–42)	39 (36–42)	39 (35–42)	0.849	0.98 (0.90–1.06)	0.564
HRCT (*n* = 140)						
HRCT bilateral involvement	135 (85.4)	15 (68.2)	120 (88.2)	0.022	0.29 (0.10–0.81)	0.018
Centrilobular micronodules	52 (34.4/32.9)	5 (22.7)	47 (36.4/34.6)	0.211	0.51 (0.18–1.48)	0.217
Cystic bronchiectasis	41 (26.6/25.9)	3 (13.6)	38 (28.8/27.9)	0.137	0.39 (0.11–1.40)	0.148
Emphysema	14 (9.9/8.9)	1 (5.0/4.5)	13 (10.7/9.6)	0.692	0.44 (0.05–3.5)	0.443
Parietal thickness	6 (4–8)	4 (3–6)	6 (4–8)	0.090	0.87 (0.73–1.03)	0.100

CF/PCD, cystic fibrosis or primary ciliary dyskinesia; OR, odds ratio; CI, confidence interval; Q1–Q3, interquartile range; ENT, ear, nose and throat; HRCT, high-resolution computed tomography. Qualitative variables are expressed as number (percentage); quantitative variables are expressed as median (Q1–Q3). * Odds ratios estimated from multivariate logistic-regression analyses. ^†^ *p* value from χ^2^, Fisher’s exact or Wilcoxon–Mann–Whitney test, as appropriate.

**Table 2:** Multivariate analysis and scoring system to predict cystic fibrosis or primary ciliary dyskinesia among adults with bronchiectasis (*n* = 158).

Characteristic	Adjusted OR (95% CI) *	*p* Value ^†^	Regression β-Coefficient ^‡^	Points
Age at symptom onset <15 years	8.69 (2.10–35.99)	0.003	2.48	5
Chronic ENT disorders	10.53 (1.26–87.57)	0.029	1.27	3
Digestive symptoms and/or dextrocardia	5.10 (1.23–21.14)	0.025	1.88	4
*P. aeruginosa* and/or *S. aureus* isolation				
None	Referent		Referent	–
*P. aeruginosa* and/or *S. aureus*	11.13 (1.34–92.2)	0.02	2.68	5

OR, odds ratio; CI, confidence interval; ENT, ear, nose and throat.* Odds ratios from multivariate unconditional logistic-regression analyses adjusted for all the variables in the model. ^†^ *p* value of the Wald test. ^‡^ β-coefficients estimated after 1000 bootstrap replications.

**Table 3:** Underlying etiologies in the validation cohort (*n* = 167).

Etiology	*n* (%)
Inflammatory disease	13 (7.8%)
Allergic bronchopulmonary aspergillosis	3
Rheumatoid arthritis	3
Sjögren’s syndrome	3
SPA	1
Lupus	1
Inflammatory bowel disease	2
Extrinsic factor	8 (4.8%)
Radiation fibrosis	6
Environmental	1
Malformation	1
Immune deficiency	9 (5.4%)
Humoral immune defect	9
Genetic disease	14 (8.4%)
CF	5
PCD	9
Miscellaneous	20 (12%)
Sarcoidosis	1
*CFTR*-related disease	1
Asthma	9
COPD	6
Isolated GERD	3
Post-infectious respiratory disease	52 (31%)
Idiopathic bronchiectasis	51 (30.5%)

CF, cystic fibrosis; PCD, primary ciliary dyskinesia; CFTR, cystic fibrosis transmembrane-conductance regulator gene; GERD, gastroesophageal reflux.

## Supplementary Materials

The following are available online at https://www.mdpi.com/article/10.3390/jcm10163478/s1, Table S1: Clinical Characteristics of the initial cohort, Table S2: Diagnosis characteristics of the 10 CF patients in the score construction group, Table S3: Performance of PICADAR score, Leigh score and our score in the validation cohort.
