# Supplementary material for: Etiological Work-Up for Adults with Bronchiectasis: A Predictive Diagnostic Score for Primary Ciliary Dyskinesia and Cystic Fibrosis
Source: J Clin Med. 2021 Aug 6;10(16):3478. doi: 10.3390/jcm10163478 (PMC8397090; doi:10.3390/jcm10163478)
Supplement: Supplementary file 1 [file jcm-10-03478-s001.zip › jcm-1312994-supplementary.pdf]

Supplementary Materials

Table S1. Clinical characteristics of the initial cohort (n=188).

Characteristic	Value
Mean age ± SD (y)	55.7±17.6
F/M sex ratio	130/58
Smoking N (%)	75 (39.9%)
Origin	
Europe	124 (66%)
Africa	12 (6.4%)
Sub-Saharan Africa	40 (21.3%)
Asia	9 (4.8%)
Caribbean/South America	3 (1.6%)
Mean age at onset of symptoms (y)	32.1± 6.1
Mean age at diagnosis of bronchiectasis (y)	44.5±22.5
Fertility	
Female (children)	103 (55%)
Male (children)	44 (23%)
History	
Measles	9 (4.8%)
Pertussis	28 (14.9%)
Tuberculosis	30 (16%)
Severe pneumonia	43 (22.9%)
History of chronic ENT symptoms	103 (54.8%)
Family history of respiratory disease	85 (45.2%)

ENT: ear nose and throat; SD: standard deviation.

Table S2 Diagnosis characteristics of the 10 CF patients in the score construction group

	Age at					
**Sex**	**1st symptom**	**Bronchiectasis Diagnosis**	**CF Diagnosis**	***CFTR* Mutation(s)**	**Sweat**	**NPD**
F	3	23	23	F508del	55	+
F	10	52	75	F508del	65	
M	50	60	72	F508del/D1152H	88	
F	6	18	44	F508del/D1152H	44	+
F	1	29	32	F508del/R117C	99	
F	6	27	36	S549N/D1155A	75	
F	27	28	34	S549N/D1155A	67	
F	12	73	73	F508del/R117H	65	
F	13	20	69	R668C/G567/D443Y	67	
M	30	30	54	S466X; R1070Q/2789+GG>A	99	

NPD: nasal potential difference; CF: cystic fibrosis; F: female; M: male.

Table S3 Performance of PICADAR score, Leigh score and our score in the validation cohort

n =136	PICADAR		Leigh		Current Score	
	≤ 5	> 5	< 2	≥ 2	≤ 8	>8
Non CF non PCD	122	0	120	2	67	55
CF	5	0	5	0	1	4
PCD	6	3	7	2	0	9

CF: cystic fibrosis; PCD: primary ciliary dyskinesia
